# Elevating VEGF-A and PDGF-BB secretion by salidroside enhances neoangiogenesis in diabetic hind-limb ischemia

**DOI:** 10.18632/oncotarget.21907

**Published:** 2017-10-13

**Authors:** Agnes Dwi Ariyanti, Julita Sisjayawan, Jing Zhang, Jian-Qi Zhang, Gui-Xue Wang, Makoto Miyagishi, Shou-Rong Wu, Vivi Kasim

**Affiliations:** ^1^ The Key Laboratory of Biorheological Science and Technology, Ministry of Education, College of Bioengineering, Chongqing University, Chongqing 400044, China; ^2^ State and Local Joint Engineering Laboratory for Vascular Implants, Chongqing 400044, China; ^3^ Molecular Composite Medicine Research Group, Biomedical Research Institute, National Institute of Advanced Industrial Science and Technology (AIST), Tsukuba 305-8566, Japan; ^4^ The 111 Project Laboratory of Biomechanics and Tissue Repair, College of Bioengineering, Chongqing University, Chongqing 400044, China

**Keywords:** hind-limb ischemia, diabetes, salidroside, skeletal muscle cells, angiogenesis

## Abstract

Hind-limb ischemia (HLI) is one of the major complication of diabetic patients. Angiogenesis potential in diabetic patients is severely disrupted, and the mechanism underlying it has not been fully elucidated, making it an obstacle for developing an efficient therapeutic angiogenesis strategy. Skeletal muscle cells, through their paracrine function, had been known to be critical for neoangiogenesis. Here we found that hyperglycemia upregulates the expression of skeletal muscle cells prolyl hydroxylase domain 3 (PHD3), which resulted in the decrease of the secretion of angiogenic factors, especially VEGF-A and PDGF-BB. We showed that treatment with salidroside, a small molecule drug, significantly suppresses PHD3 expression and increases VEGF-A and PDGF-BB secretion from skeletal muscle cells, which in turn enhances the proliferation and migration potentials of endothelial and smooth muscle cells. Finally, we demonstrated that intramuscular injection of salidroside into the ischemic hind limbs of diabetic HLI model mice could efficiently induce neoangiogenesis and blood perfusion recovery. Thus, our novel findings not only reveal the effects of hyperglycemia on the angiogenesis potential of skeletal muscle cells and the mechanism underlying it, but also provides a novel finding suggesting that salidroside might be a potential small molecule drug for diabetic HLI.

## INTRODUCTION

Diabetes mellitus is a chronic metabolic disorder that is characterized by hyperglycemia, which could result in serious damages to the heart, eyes, kidneys, nerves and blood vessels [1, 2]. Diabetic vascular complications are the greatest contributors to diabetes-associated morbidity and mortality [3–5]. Diabetic patients are at excess risk of developing hind-limb ischemia (HLI), in which the blood supply to the limbs is gradually reduced due to the narrowing or occlusions of the arteries [6]. The prognosis of diabetic HLI patients is significantly worse than the non-diabetic ones: amputation risk of diabetic patients with HLI is 5 times higher, while the mortality rate is more than 3 times higher [6]. Conventional treatments, such as surgical approach using cathether- or stent-based revascularization is not effective for diabetic HLI patients, as they usually have large wound surface and high recurrence rate [7].

On the other hand, therapeutic angiogenesis, which aims to induce the formation of new blood vessels to compensate the lack of oxygen and nutrients supply to the ischemic tissues, has been considered as a potential therapeutic strategy for HLI patients, including those who have been assumed as “no choice” for surgical based-therapy [8]. However, despite that several promising therapeutic angiogenesis strategies in HLI patients without diabetes had been reported, effective angiogenesis induction in diabetic HLI patients remains an obstacle. Hyperglycemia systematically impairs endogenous angiogenesis potential, as it induces aberrant expression of various angiogenesis factors, such as hypoxia-inducible factor 1 (HIF1)-α [9], vascular endothelial growth factor (VEGF) [10, 11] and platelet-derived growth factor (PDGF)-BB [12], resulted in the disruption of endogenous angiogenesis pathways. Furthermore, the detail mechanism of the impaired angiogenesis potential induces by hyperglycemia remains unknown, rendering the development of potential, effective therapeutic strategy.

Skeletal muscle is the largest secretory organ in the body. It has currently become an attractive target for therapeutic angiogenesis, as it could secrete various angiogenic factors [8, 13, 14]. These secreted factors could promote cell–cell communication between skeletal muscle cells and cells forming mature vessels, *i.e.*, endothelial and smooth muscle cells, and in turn affect neoangiogenesis [13, 14]. Indeed, enhancing the paracrine function of skeletal muscle cells had been shown as a potential strategy for HLI [14, 15]. However, whether skeletal muscle cells-based therapeutic angiogenesis is effective and sufficient for inducing functional neoangiogenesis in diabetic HLI patients remains unknown.

In this study, we found that hyperglycemia induced the accumulation of prolyl hydroxylase domain 3 (PHD3) in the skeletal muscle cells. PHD3 has been known to enhance protein degradation of HIF-1α, a key master for the induction of various angiogenesis factors [16, 17]. We showed that PHD3 inhibition using small molecule drug inhibitor, salidroside, restored skeletal muscle cells proliferation and angiogenic factors secretion potentials suppressed by hyperglycemia. Treatment with salidroside enhanced skeletal muscle cells–endothelial and/or smooth muscle cells communication mediated by VEGF-A and PDGF-BB, and in turn, enhanced the proliferation and migration potentials of endothelial and smooth muscle cells under hyperglycemia. Subsequently, we revealed that intramuscular treatment of salidroside could effectively induce the formation of mature blood vessels and the blood perfusion recovery in diabetic HLI model mice.

## RESULTS

### Hyperglycemia suppresses the expression of angiogenic factors in skeletal muscle cells via inducing PHD3 accumulation

**Figure 1:**
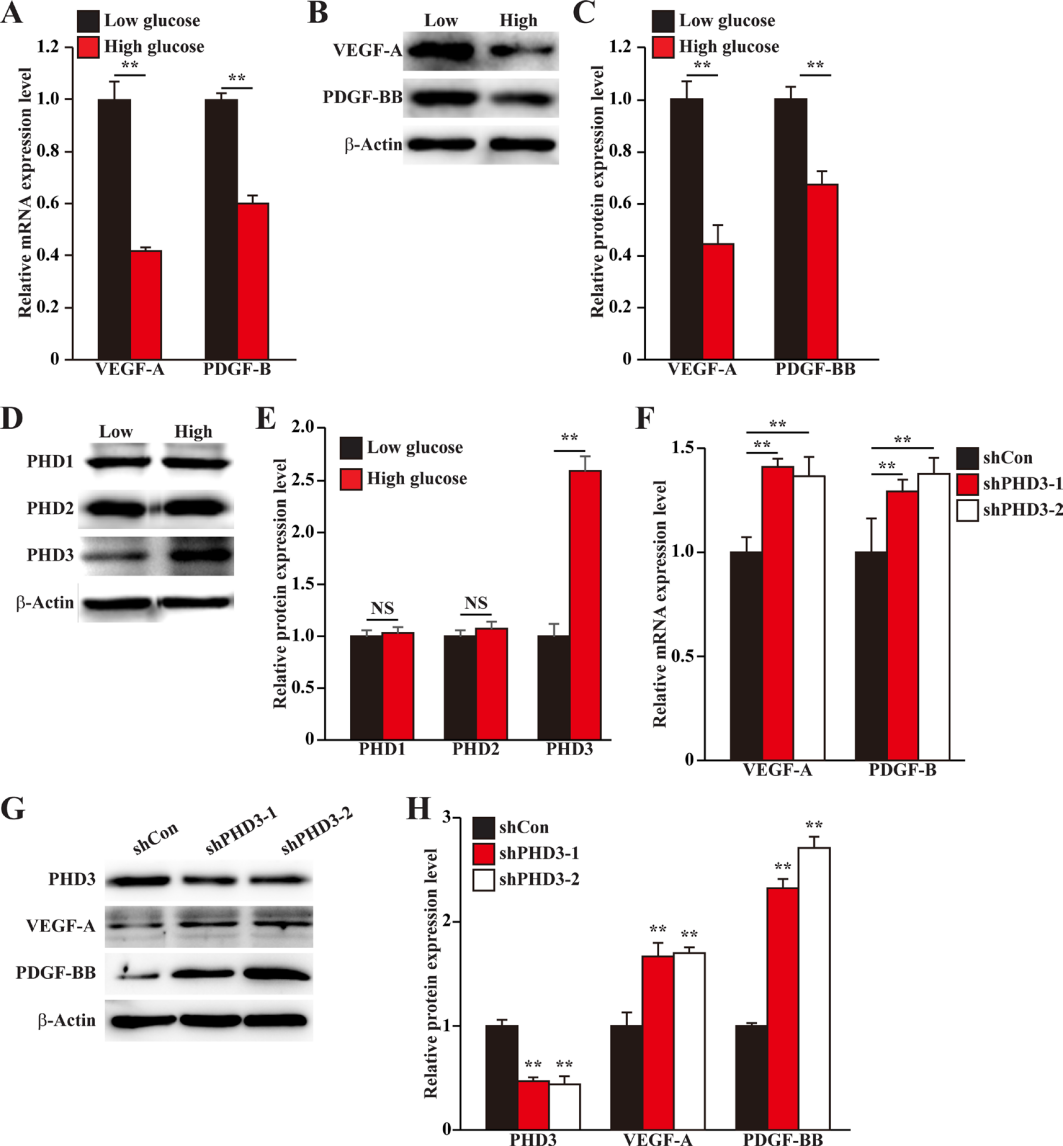
Hyperglycemia suppresses angiogenic factors expression in skeletal muscle cells by inducing PHD3 protein accumulation (**A–C**) The expression levels of angiogenic factors in C2C12 cells cultured under hyperglycemia: (A) mRNA expression levels, as examined by quantitative RT-PCR (qPCR); (B) Protein expression levels, as examined by western blotting; and (C) quantification of protein expression levels. (**D, E**) Protein expression levels of PHD family in C2C12 cells cultured under hyperglycemia, as determined using western blotting: (D) representative images, and (E) quantification of protein expression levels. (**F–H**) Expression levels of angiogenic factors in PHD3-silenced C2C12 cells cultured under hyperglycemia: (F) mRNA expression levels, as examined using qPCR; (G) protein expression levels, as determined using western blotting; and (H) quantification of protein expression levels. Cells cultured under low glucose condition were used as control. All experiments were done under hypoxia. β-Actin was used for normalization in qPCR, and as a loading control in western blotting. Quantification data were shown as relative to that of control, and expressed as mean ± S.D. (*n* = 3 from three independent experiments). NS: not significant; ^**^P < 0.01; Low: low glucose; High: high glucose.

Skeletal muscle cells could express various angiogenic factors, and thus play a pivotal role in angiogenesis. To mimic the pathological condition of diabetic HLI, we established hypoxic, hyperglycemic condition as described previously [18], and investigated the effect of hyperglycemic condition on the expression levels of angiogenic factors in skeletal muscle cells. As shown in Figure 1A, the mRNA expression levels of VEGF-A and PDGF-B in murine myoblast cell line C2C12 were significantly suppressed under hyperglycemia. The protein expression levels of these angiogenic factors showed similar tendency: hyperglycemia downregulated the levels of VEGF-A and PDGF-BB proteins in C2C12 cells (Figure 1B, 1C).

PHD family had been known as a crucial factor in regulating the expression of various angiogenic factors [19–21]. Thus, we next examined the effect of hyperglycemia on the expression levels of PHD family, *i.e.* PHD1, PHD2 and PHD3, in C2C12 cells. We found that while the protein expression levels of PHD1 and PHD2 were not significantly affected, hyperglycemia robustly induced PHD3 protein expression level (Figure 1D, 1E). To investigate the relationship between the hyperglycemia-induced PHD3 protein accumulation and the reduction of angiogenic factors expressions in skeletal muscle cells, we knocked down PHD3 expression using short hairpin RNA (shRNA) expression vectors. As shown in Supplementary Figure 1, both shRNA expression vectors targeting two different sites of PHD3, *i.e.*, shPHD3-1 and shPHD3-2, could suppress the mRNA expression level of PHD3 for more than 60%. Silencing of PHD3 grossly elevated the mRNA expression levels of VEGF-A and PDGF-B under hyperglycemia (Figure 1F). Furthermore, the effect of PHD3-silencing on VEGF-A and PDGF-BB proteins showed similar tendency: PHD3-silencing stimulated VEGF-A and PDGF-BB protein expression levels under hyperglycemia (Figure 1G, 1H). Thus, hyperglycemia decreases the expression levels of angiogenic factors in skeletal muscle cells through, at least partly, the accumulation of PHD3 protein.

### Salidroside improves angiogenic potentials of skeletal muscle cells under hyperglycemia through PHD3 inhibition

**Figure 2:**
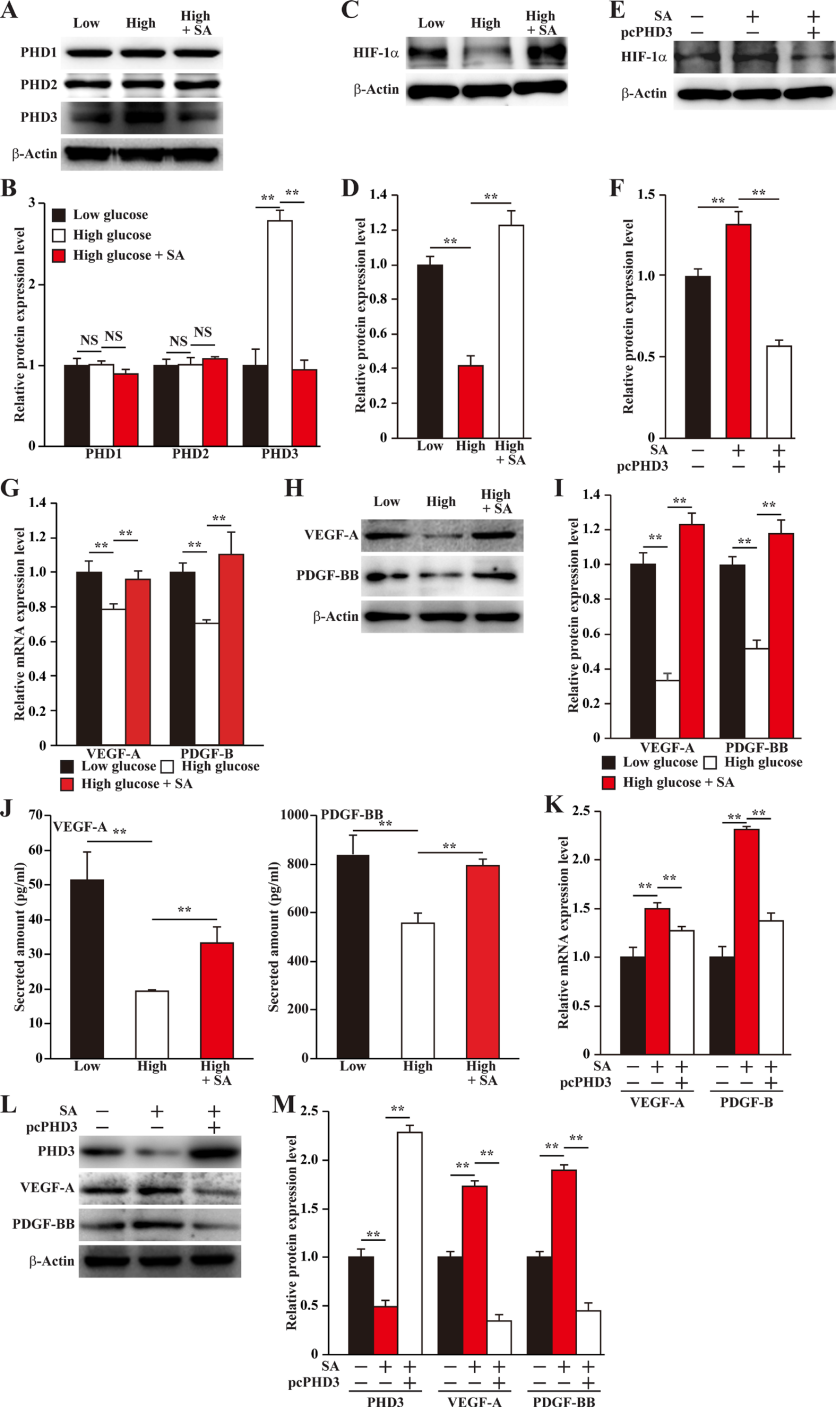
Salidroside restores the expression of skeletal muscle cells angiogenic factors repressed by hyperglycemia (**A, B**) Protein expression levels of PHD family in the C2C12 cells treated with salidroside and cultured under hyperglycemia, as determined by western blotting: (A) representative images; and (B) quantification of protein expression levels. (**C, D**) Protein expression level of HIF-1α in C2C12 cells treated with salidroside and cultured under hyperglycemia, as determined by western blotting: (C) representative images; and (D) quantification of protein expression level. (**E, F**) Protein expression level of HIF-1α in the PHD3-overexpressing C2C12 cells cultured under hyperglycemia and treated with salidroside, as determined by western blotting: (E) representative images; and (F) quantification of protein expression level. (**G–I**) Expression levels of VEGF-A and PDGF-BB in C2C12 cells treated with salidroside and cultured under hyperglycemia: (G) mRNA expression levels, as analyzed by quantitative RT-PCR (qPCR); (H) protein expression levels, as examined by western blotting; and (I) quantification of protein expression levels. (**J**) Secretion levels of VEGF-A (left panel) and PDGF-BB (right panel) from C2C12 cells treated with salidroside and cultured under hyperglycemia, as analyzed by ELISA. (**K–M**) Expression levels of VEGF-A and PDGF-BB in the PHD3-overexpressing C2C12 cells cultured under hyperglycemia and treated with salidroside: (K) mRNA expression levels, as quantified by qPCR; (L) protein expression levels, as examined by western blotting; and (M) quantification of protein expression levels. All experiments were done under hypoxia, and cells cultured under low glucose condition or cells transfected with pcCon and treated with PBS were used as controls. β-Actin was used for normalization in qPCR, and as a loading control in western blotting. Quantification data were shown as relative to that of control, and expressed as mean ± S.D. (*n* = 3 from three independent experiments). ^**^P < 0.01; Low: low glucose; High: high glucose; SA: salidroside.

Our very recent report found that under normoglycemia, salidroside could specifically suppress PHD3 [14]. Therefore, we next investigated whether salidroside could suppress the aberrant expression of skeletal muscle cells PHD3 under hyperglycemia. Our results showed that salidroside could robustly suppress PHD3 protein accumulation induced by hyperglycemia (Figure 2A, 2B). Given that PHD3 increases the degradation of HIF-1α protein, a key factor of angiogenesis that regulates multiple angiogenic factors including VEGF-A and PDGF-BB [16, 22, 23], we then examined whether the suppressive effect of salidroside on PHD3 expression was able to induce HIF-1α protein accumulation. Indeed, we found that salidroside treatment restored HIF-1α protein accumulation, which was suppressed by hyperglycemia (Figure 2C, 2D); while overexpression of PHD3 cancelled this effect (Figure 2E, 2F). Concomitantly, salidroside could restore the expression levels of VEGF-A and PDGF-BB repressed by hyperglycemia (Figure 2G–2I). Consistent with these, the levels of VEGF-A and PDGF-BB secreted from C2C12 cells also decreased upon exposure to hyperglycemia, while treatment with salidroside conspicuously enhanced them (Figure 2J). These results suggested the possibility that salidroside might be able to enhance angiogenic factors expression by suppressing hyperglycemia-induced PHD3 accumulation. To further confirm this regulatory pathway, we analyzed the angiogenic factors expression levels in PHD3-overexpressing C2C12 cells treated with salidroside, and cultured under hyperglycemic condition. From the results shown in Figure 2K–2M, it is obvious that in the C2C12 cells overexpressing PHD3, the effect of salidroside on stimulating the expression levels of VEGF-A and PDGF-BB was cancelled. Therefore, we found, for the first time, that salidroside could cancelled the hyperglycemia-induced PHD3 accumulation in the skeletal muscle cells, which resulted in the restoration of HIF-1α protein accumulation and angiogenic factors expression, especially VEGF-A and PDGF-BB.

**Figure 3:**
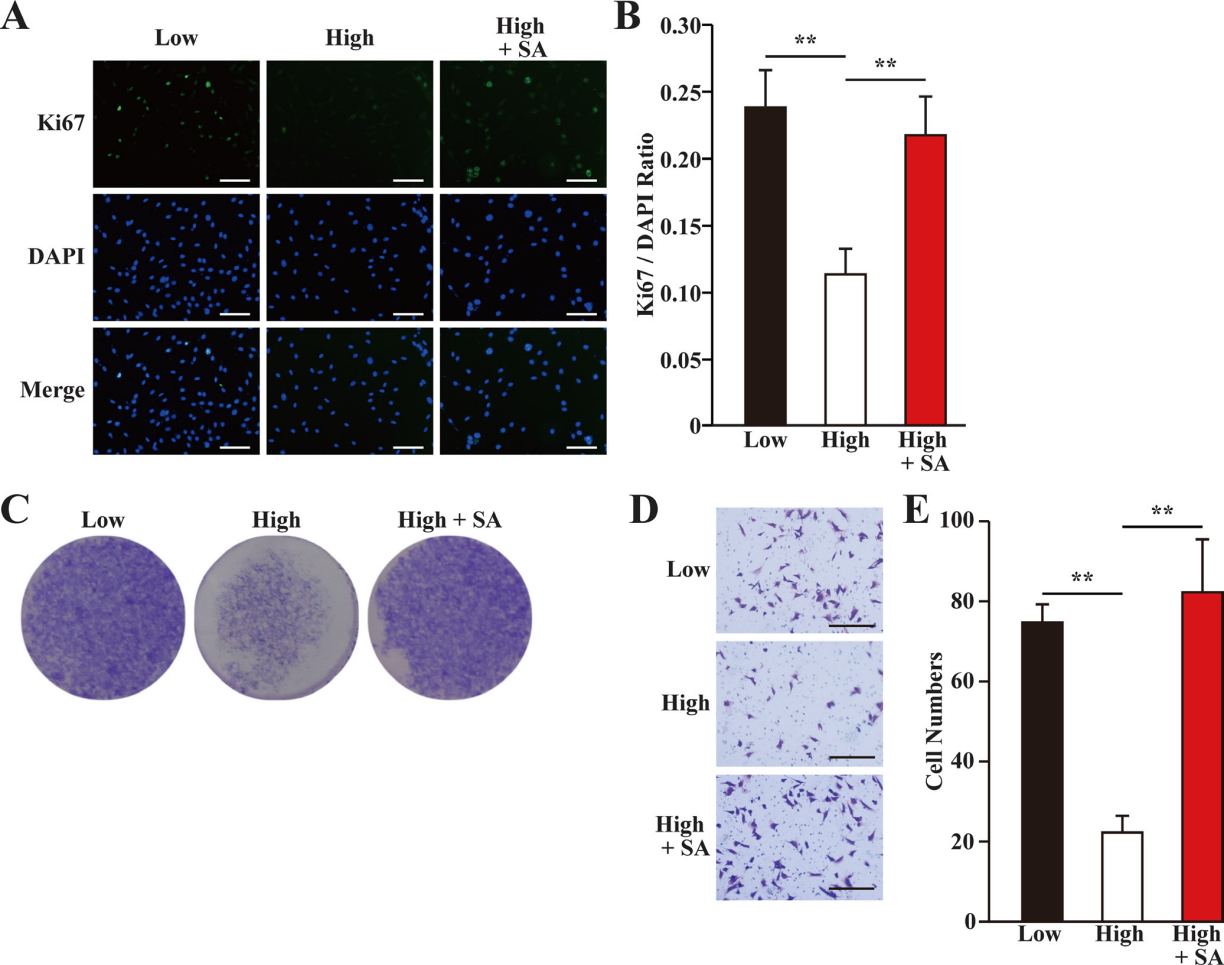
Salidroside restores skeletal muscle cells proliferation and migration potentials suppressed by hyperglycemia (**A, B**) The percentage of proliferative C2C12 cells treated with salidroside and cultured under hyperglycemia, as analyzed using Ki67 staining: (A) representative images (scale bars: 200 μm); and (B) quantification of the ratio of Ki67 positive cells to DAPI positive cells. (**C**) The number of C2C12 cells treated with salidroside and cultured under hyperglycemia, as analyzed using crystal violet staining. (**D, E**) The migration potential of C2C12 cells treated with salidroside and cultured under hyperglycemia, as examined using transwell chamber assay: (D) representative images (scale bars: 100 μm); and (E) quantification of migrated cells. All experiments were done under hypoxia. Data shown are representative from three independent experiments. Quantification data were expressed as mean ± S.D. (*n* = 6). ^**^P < 0.01; SA: salidroside.

VEGF-A had been reported to stimulate the proliferation and migration of skeletal muscle cells [24]. Indeed, we showed that hyperglycemia grossly suppressed the proliferation potential of skeletal muscle cells, while salidroside treatment could restored it (Figure 3A–3C). Similarly, the migration potential of skeletal muscle cells significantly decreased under hyperglycemia, while treatment with salidroside induced it (Figure 3D, 3E). Together, these results showed that while hyperglycemia suppressed the angiogenic factors secretion, proliferation and the migration potentials of C2C12 cells, salidroside significantly enhanced these biological functions, indicating that salidroside might be able to promote neoangiogenesis under hyperglycemia.

### Secretome from salidroside-treated skeletal muscle cells promotes proliferation and migration potentials of endothelial and smooth muscle cells

**Figure 4:**
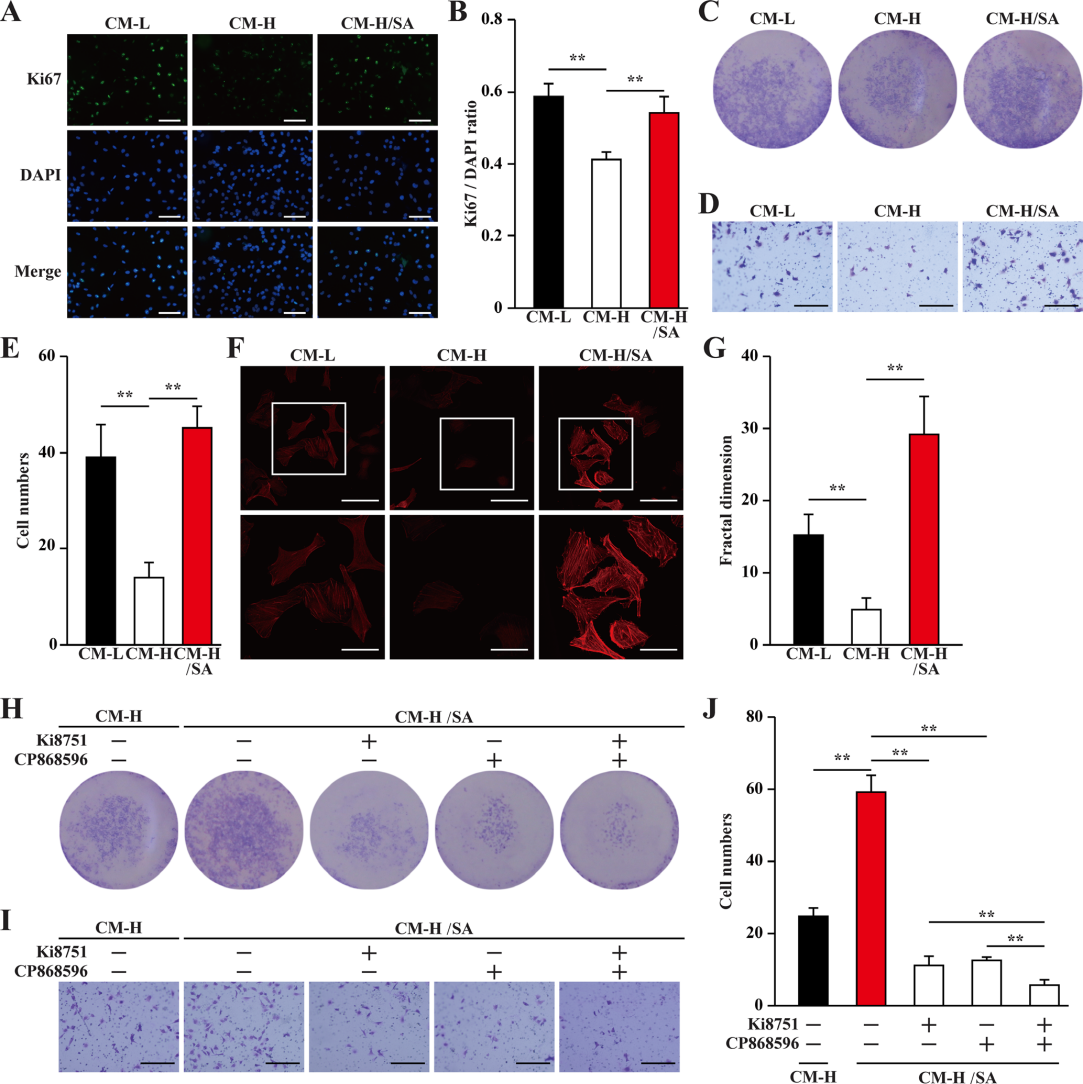
Salidroside promotes endothelial cells proliferation and migration potentials via skeletal muscle cells secretome (**A, B**) The percentage of proliferative HUVECs cultured with indicated conditioned media, as analyzed by Ki67 staining: (A) representative images (scale bars: 200 μm); and (B) quantification of the ratio of Ki67 positive cells to DAPI positive cells. (**C**) The number of HUVECs cells cultured with indicated conditioned media, as analyzed by crystal violet staining. (**D, E**) The mobility of HUVECs cultured with indicated conditioned media, as examined by transwell chamber assay: (D) representative images (scale bars: 100 μm); and (E) quantification of migrated cells. (**F, G**) Morphological changes of F-Actin, as examined by phalloidin staining: (F) representative images (scale bars: 100 μm for upper panels and 50 μm for lower panels), lower panels showed the enlarged images of the cropped part in the upper panels; (G) quantification analysis of fractal dimension. (**H**) The number of HUVECs cultured with CM-H/SA and indicated inhibitors, as analyzed by crystal violet staining. (**I, J**) The mobility of HUVECs cultured with CM-H/SA and indicated inhibitors, as examined by transwell chamber assay: (I) representative images (scale bars: 100 μm); and (J) quantification of migrated cells. All experiments were done under hypoxia. Data shown are representative from three independent experiments. Quantification data were expressed as mean ± S.D. (*n* = 6). ^**^P < 0.01. CM-L: conditioned medium collected from C2C12 cells treated with PBS and cultured under low glucose condition; CM-H: conditioned medium collected from C2C12 cells treated with PBS and cultured under hyperglycemia; and CM-H/SA: conditioned medium collected from C2C12 cells treated with salidroside and cultured under hyperglycemia; Ki8751: VEGFR inhibitor; CP868596: PDGFR-β inhibitor.

Mature blood vessels are formed by endothelial cells covered by smooth muscle cells. Paracrine signals, especially those of angiogenic factors such as VEGF-A and PDGF-BB from skeletal muscle cells, could effectively affect the biological functions of endothelial and smooth muscle cells, and subsequently, induce neoangiogenesis. Thus, we examine whether the upregulatory effect of salidroside on the secretion of angiogenic factors from skeletal muscle cells under hyperglycemia is sufficient to affect endothelial and smooth muscle cells. For this purpose, we collected the conditioned medium from C2C12 cells treated with salidroside and cultured under hyperglycemia (CM-H/SA), as well as from C2C12 cells cultured under low glucose or hyperglycemic conditions (CM-L and CM-H, respectively). It is noteworthy that salidroside could not directly affect the proliferation and migration of endothelial cells (human umbilical vein endothelial cells, HUVECs, Supplementary Figure 2A–2C), and smooth muscle cells (MOVAS cells, Supplementary Figure 2D–2F). Furthermore, to avoid carry over of salidroside, we washed the C2C12 cells after 24 h treatment with salidroside and prior to further incubation under hyperglycemia. We found that compared with those cultured with CM-L, HUVECs cultured with CM-H showed significantly reduced Ki67 positive cells ratio; while the ratio of Ki67 positive in HUVECs cultured with CM-H/SA was similar with those cultured with CM-L (Figure 4A, 4B). The results of crystal violet staining further confirmed this tendency: compared to those cultured with CM-H, culture with CM-H/SA resulted in a robust increase in the number of HUVECs (Figure 4C). These results suggested that CM-H/SA could restore the decrase of the endothelial cells proliferation rate caused by the secretome from skeletal muscle cells cultured under hyperglycemia.

Furthermore, the results of transwell chamber assay also showed that CM-H robustly suppressed HUVECs migration potential for more than two-folds; however, this suppression was cancelled when the CM-H/SA was used (Figure 4D, 4E). Moreover, using phalloidin staining and quantification by fractal dimension analysis to measure the F-Actin formed by polymerization of G-Actin [25], we revealed that the differences of the migration potential was most plausibly due to the difference in the actin filament polymerization potential, as the pseudopodia and F-Actin polymerization was robustly decreased when CM-H was used, and grossly increased when CM-H/SA was used (Figure 4F, 4G).

VEGF-A and PDGF-BB are crucial angiogenic factors. VEGF-A is essential in promoting the proliferation and migration of endothelial cells, which form the vasculature structures, while PDGF-BB is essential for enhancing both the migration and proliferation potentials of smooth muscle cells and endothelial cells [26–29]. To further confirm the roles of VEGF-A and PDGF-BB in increasing the proliferation and migration potentials of endothelial cells induced by the secretome of salidroside-treated skeletal muscle cells, we added the inhibitors of their receptors (Ki8751 for VEGFR or CP868596 for PDGFR-β) in the conditioned medium. We found that inhibition of either VEGF-A/VEGFR or PDGF-BB/ PDGFR-β pathway significantly decreased the effect of CM-H/SA on the proliferation and migration potentials of endothelial cells; while blocking both pathways almost diminished these potentials (Figure 4H–4J).

**Figure 5:**
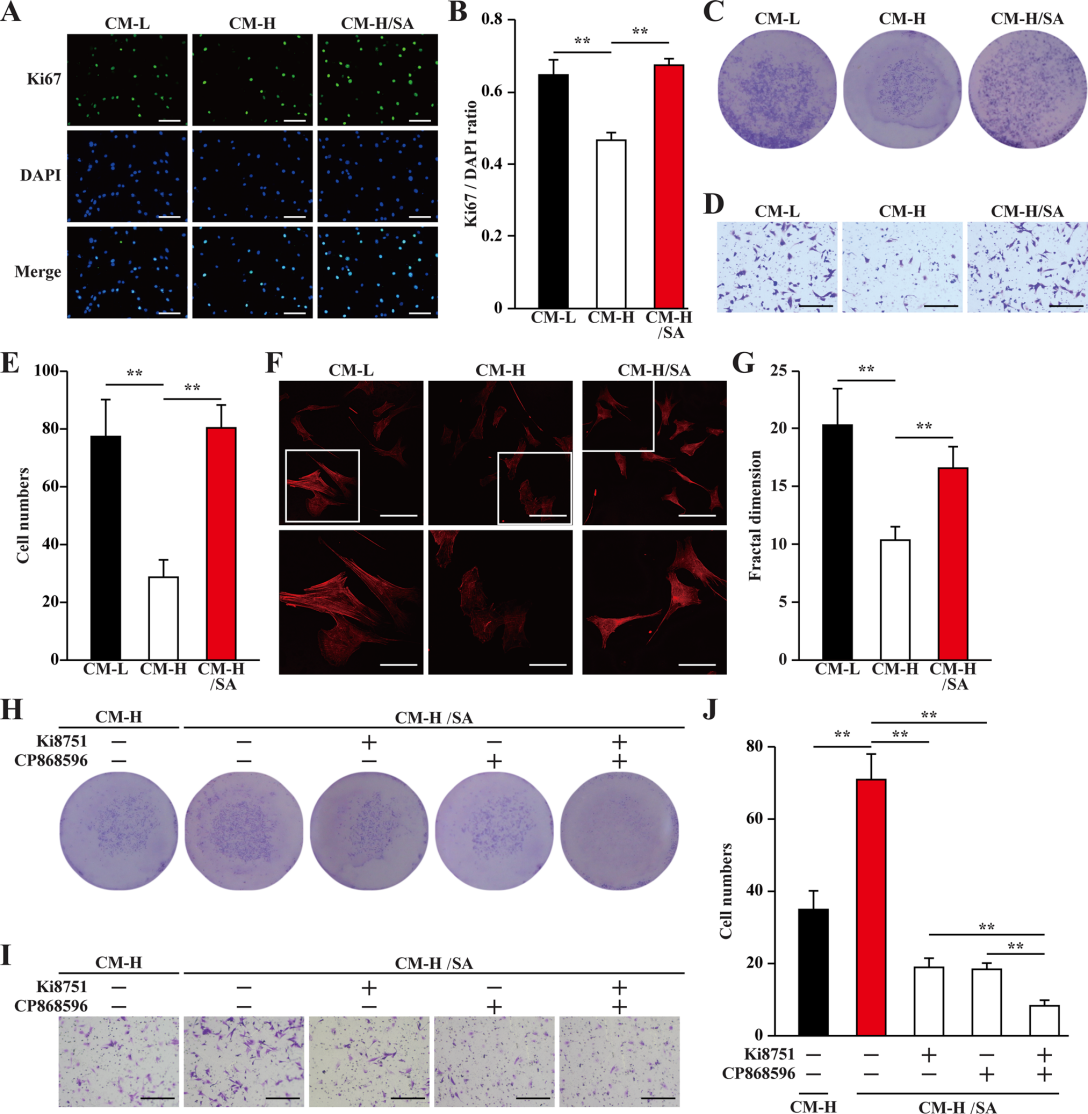
Salidroside promotes smooth muscle cells proliferation and migration potentials via skeletal cells secretome (**A, B**) The proliferation of MOVAS cells cultured with indicated conditioned media, as analyzed by Ki67 staining: (A) representative images (scale bars: 200 μm); and (B) quantification of the ratio of Ki67 positive cells to DAPI positive cells. (**C**) The number of MOVAS cells cultured with indicated conditioned media, as analyzed by crystal violet. (**D, E**) The mobility of MOVAS cells cultured with indicated conditioned media was examined using transwell chamber assay: (D) representative images (scale bars: 100 μm); and (E) quantification of migrated cells. (**F, G**) Morphological changes of F-Actin, as examined by phalloidin staining: (F) representative images (scale bars: 100 μm for upper panels and 50 μm for lower panels), lower panels showed the enlarged images of the cropped part in the upper panels; (G) quantification analysis of fractal dimension. (**H**) The number of MOVAS cells cultured with CM-H/SA and indicated inhibitors, as analyzed by crystal violet staining. (**I, J**) The mobility of MOVAS cells cultured with CM-H/SA and indicated inhibitors, as examined by transwell chamber assay: (I) representative images (scale bars: 100 μm); and (J) quantification of migrated cells. All experiments were done under hypoxia. Data shown are representative from three independent experiments. Quantification data were expressed as mean ± S.D. (*n* = 6). ^**^P < 0.01. CM-L: conditioned medium collected from C2C12 cells treated with PBS and cultured under low glucose condition; CM-H: conditioned medium collected from C2C12 cells treated with PBS and cultured under hyperglycemia; and CM-H/SA: conditioned medium collected from C2C12 cells treated with salidroside and cultured under hyperglycemia; Ki8751: VEGFR inhibitor; CP868596: PDGFR-B inhibitor.

We next investigated the effect of CM-L, CM-H and CM-H/SA on MOVAS cells. Similar to their effects on endothelial cells, MOVAS cells proliferation was also abrogated when cultured with CM-H, but this abrogation was not observed when the cells were incubated with CM-H/SA (Figure 5A–5C). Furthermore, the same tendencies were also observed in the migration potential and F-Actin polymerization: CM-H disrupted the migration potential and F-Actin polymerization, while CM-H/SA grossly improved them (Figure 5D–5G). Similar to endothelial cells, addition of VEGFR or PDGFR-β inhibitors robustly suppressed salidroside-induced smooth muscle cells proliferation and migration, while addition of both inhibitors almost totally cancelled these effects (Figure 5H–5J).

Together, these results suggested that the effect of conditioned medium from skeletal muscle cells on the proliferation and migration potentials of endothelial and smooth muscle cells showed positive correlations with the angiogenic potentials of skeletal muscle cells, *i.e.*, the potentials were decrease under hyperglycemia, and restored upon treatment with salidroside. At the same time, these results also revelaed the importance of VEGF-A/VEGFR and PDGF-BB/PDGFR-β pathways in the salidroside-induced, skeletal muscle cells secretome-mediated stimulation of endothelial and smooth muscle cells proliferation and migration potentials.

### PHD3 overexpression cancels paracrine function of salidroside-treated skeletal muscle cells under hyperglycemia

**Figure 6:**
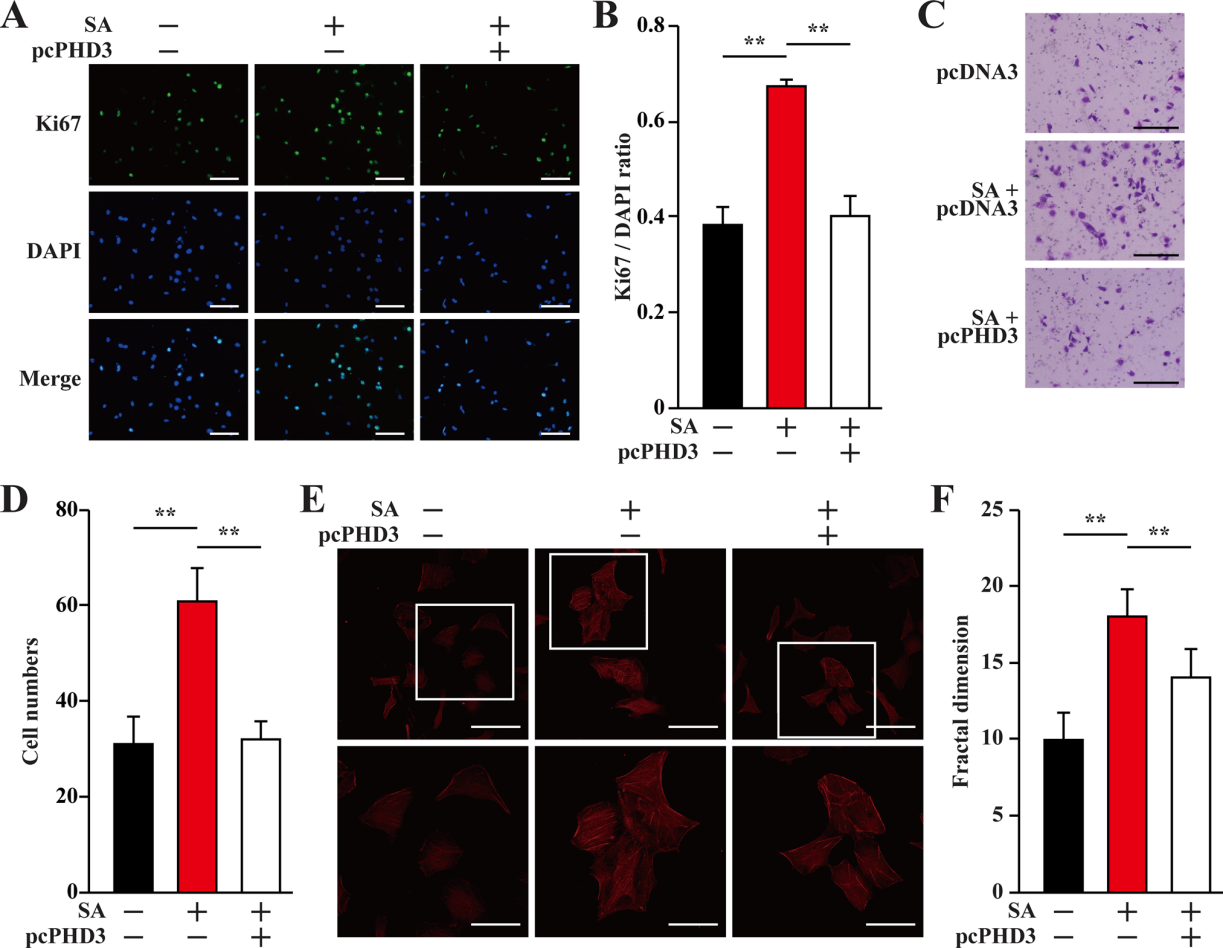
Salidroside enhances endothelial cells proliferation and migration potentials by suppressing hyperglycemia-induced skeletal muscle cells PHD3 accumulation (**A, B**) The proliferation of HUVECs cultured with indicated conditioned media, as analyzed by Ki67 staining: (A) representative images (scale bars: 200 μm); and (B) quantification of the ratio of Ki67 positive cells to DAPI positive cells. (**C, D**) The mobility of HUVECs cultured with indicated conditioned media, as examined by transwell chamber assay: (C) representative images (scale bars: 100 μm); and (D) quantification of migrated cells. (**E, F**) Morphological changes of F-Actin, as determined by phalloidin staining: (E) representative images (scale bars: 100 μm for upper panels and 50 μm for lower panels), lower panels showed the enlarged images of the cropped part in the upper panels; (F) quantification analysis of fractal dimension. Conditioned media were collected from C2C12 cells transfected with pcCon or pcPHD3, treated with PBS or salidroside and cultured under hyperglycemia. All experiments were done under hypoxia. Data shown are representative from three independent experiments. Quantification data were expressed as mean ± S.D. (*n* = 6). ^**^P < 0.01; pcCon: pcDNA3.1(+); SA: salidroside.

As shown in Figure 1, hyperglycemia disrupted the expressions of angiogenic factors in skeletal muscle cells through inducing PHD3 protein accumulation. On the other hand, salidroside suppressed the accumulation of PHD3 induced by hyperglycemia, resulting in the increase of skeletal muscle cells VEGF-A and PDGF-BB secretion. To examine the role of PHD3 in promoting the proliferation and migration potentials of endothelial and smooth muscle cells induced by salidroside-treated skeletal muscle cells secretome under hyperglycemia, we collected conditioned media from C2C12 cells cultured under hyperglycemia; as well as conditioned media from C2C12 cells or C2C12 cells overexpressing PHD3, treated with salidroside and cultured under hyperglycemia. As shown in Figure 6A and 6B, PHD3 overexpression significantly decreased the effect of salidroside mediated by C2C12 cells secretome on endothelial cells proliferation. Similarly, both the migration and F-Actin polymerization potentials induced by the conditioned medium from C2C12 cells treated with salidroside were also robustly repressed when the conditioned medium was obtained from C2C12 cells overexpressing PHD3 (Figure 6C–6F).

**Figure 7:**
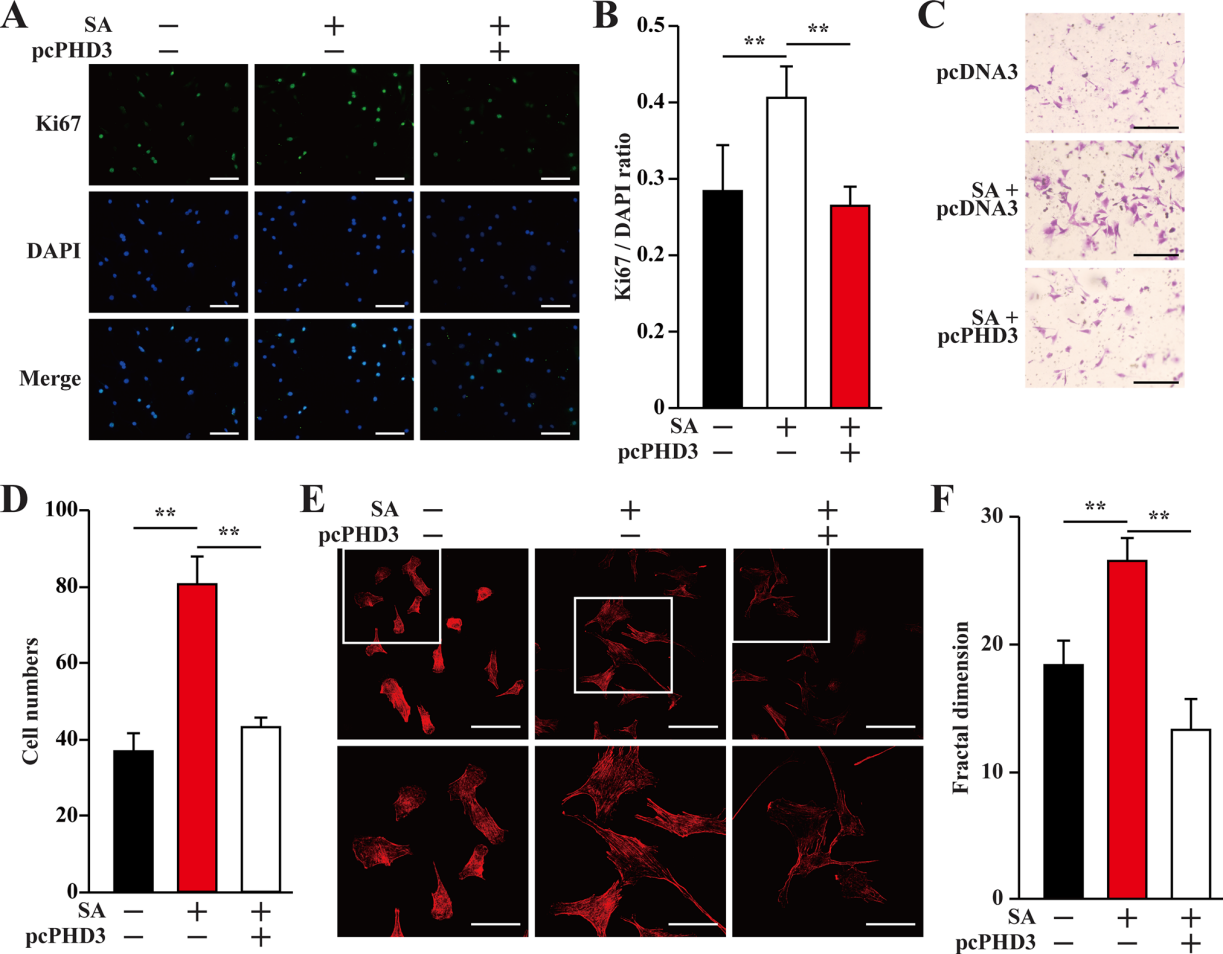
Salidroside enhances smooth muscle cells proliferation and migration potentials by suppressing hyperglycemia-induced skeletal muscle cells PHD3 accumulation (**A, B**) The proliferation of MOVAS cells cultured with indicated conditioned media, as analyzed by Ki67 staining: (A) representative images (scale bars: 200 μm); and (B) quantification of the ratio of Ki67 positive cells to DAPI positive cells. (**C, D**) The mobility of MOVAS cells cultured with conditioned media, as examined by transwell chamber assay: (C) representative images (scale bars: 100 μm); and (D) quantification of migrated cells. (**E, F**) Morphological changes of F-Actin, as examined using phalloidin staining: (E) representative images (scale bars: 100 μm for upper panels and 50 μm for lower panels), lower panels showed the enlarged images of the cropped part in the upper panels; (F) quantification analysis of fractal dimension. Conditioned media were collected from C2C12 cells transfected with pcCon or pcPHD3, treated with PBS or salidroside and cultured under hyperglycemia. All experiments were done under hypoxia. Data shown are representative from three independent experiments. Quantification data were expressed as mean ± S.D. (*n* = 6). ^**^P < 0.01; pcCon: pcDNA3.1(+); SA: salidroside.

Consistent with HUVECs, the same tendencies were also showed by MOVAS cells. When the conditioned medium used was collected from C2C12 cells overexpressing PHD3, the effects of the conditioned medium from salidroside-treated C2C12 cells on restoring the proliferation, migration and F-Actin polymerization potentials of MOVAS cells under hyperglycemia were cancelled (Figure 7A–7F). Thus, it is obvious that salidroside increased the angiogenic factors-mediated cell–cell communications between skeletal muscle cells and endothelial and/or smooth muscle cells, which were suppressed under hyperglycemia, by suppressing PHD3 protein accumulation in skeletal muscle cells.

### Salidroside promotes blood perfusion recovery and neoangiogenesis in diabetic HLI model mice

**Figure 8:**
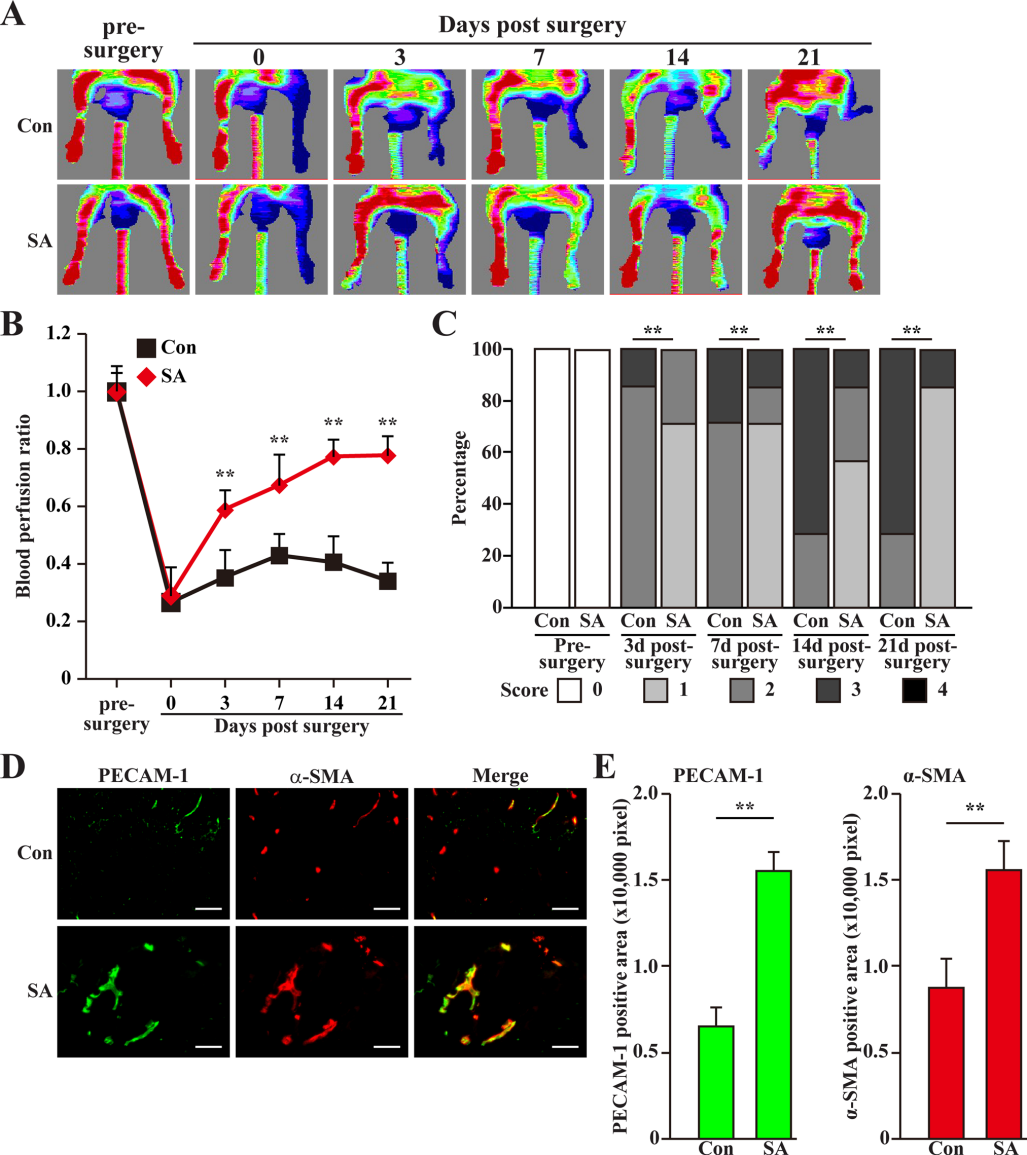
Salidroside promotes blood perfusion recovery by inducing neoangiogenesis in diabetic HLI model mice (**A**) Representative of Laser Doppler Perfusion Images showing blood perfusion in the ischemic and non-ischemic hind limb of diabetic HLI model mice treated with PBS (upper panels) or salidroside (lower panels). (**B**) The blood perfusion ratio of ischemic hind limb to non-ischemic hind limb. Data were shown as mean ± S.D. (*n* = 7 per group). (**C**) Assessment test of the ischemic hind limb morphologies of diabetic HLI model mice treated by salidroside or PBS at indicated times (*n* = 7 per group, 0 = no difference from the non-ischemic hind limb, 1 = mild discoloration, 2 = moderate discoloration, 3 = severe discoloration, subcutaneous tissue loss, or necrosis, and 4 = amputation). (**D, E**) Immunohistochemistry against PECAM-1 (green) and α-SMA (red) in the gastrocnemius muscle of the ischemic hind limbs of diabetic HLI model mice treated by either salidroside or PBS at 21 days post-surgery: (D) representative images (scale bars: 100 μm); (E) quantification of PECAM-1 (left panel) and α-SMA positive (right panel) areas. Data shown are representative from three independent experiments. Quantification data were shown as mean ± S.D. (*n* = 6). ^**^P < 0.01 (control *versus* salidroside-treated); Con: animals treated with PBS; SA: animals treated with salidroside.

Finally, we investigated the possibility of using salidroside for treating diabetic HLI. For this purpose, we induced diabetic condition in C57BL/6 mice using high fat diet and streptozotocin [30]. Mice with blood glucose more than 16.7 mmol/L were then subjected to femoral artery excision and intramuscular injection of salidroside [14]. As shown in Figure 8A, salidroside treatment resulted in a gradual blood perfusion recovery started from the third day after surgery. At three weeks after surgery, the blood perfusion in the ischemic hind limb of mice treated with salidroside had recovered to more than 70% of the non-ischemic contralateral limb; however, for those treated with PBS, the blood perfusion ratios were kept at the level less than 40% until the end of the experiment (Figure 8B). Consistently, salidroside therapeutic effect on diabetic HLI was also reflected by the ischemic damage assessment results: at the end of the experiment, most of the mice treated with salidroside scored 1, while more than half of the mice treated with PBS scored 3 (Figure 8C).

To further investigate whether or not the effect of salidroside on the blood perfusion recovery was due to the induction of neoangiogenesis, we performed immunohistochemistry against endothelial and smooth muscle cells markers: platelet endothelial cell adhesion molecule 1 (PECAM-1) and alpha-smooth muscle actin (α-SMA), respectively. As shown in Figure 8D and 8E, compared to those treated with PBS, we observed significant increase in the number of PECAM-1 and α-SMA positive cells in the ischemic hind limb of the diabetic HLI model mice treated with salidroside. Consistently, the number of PECAM-1 and α-SMA double positive cells forming vessel structure was also robustly upregulated, indicating the increase of the number of mature vessel (Figure 8D, merge). Furthermore, it is also noteworthy that compared to those treated with PBS, gastrocnemius muscle cells obtained from diabetic HLI model mice treated with salidroside showed a more intact tissue structure (Supplementary Figure 3), suggesting that salidroside might also protect the skeletal muscle tissues from damages caused by hyperglycemia.

**Figure 9:**
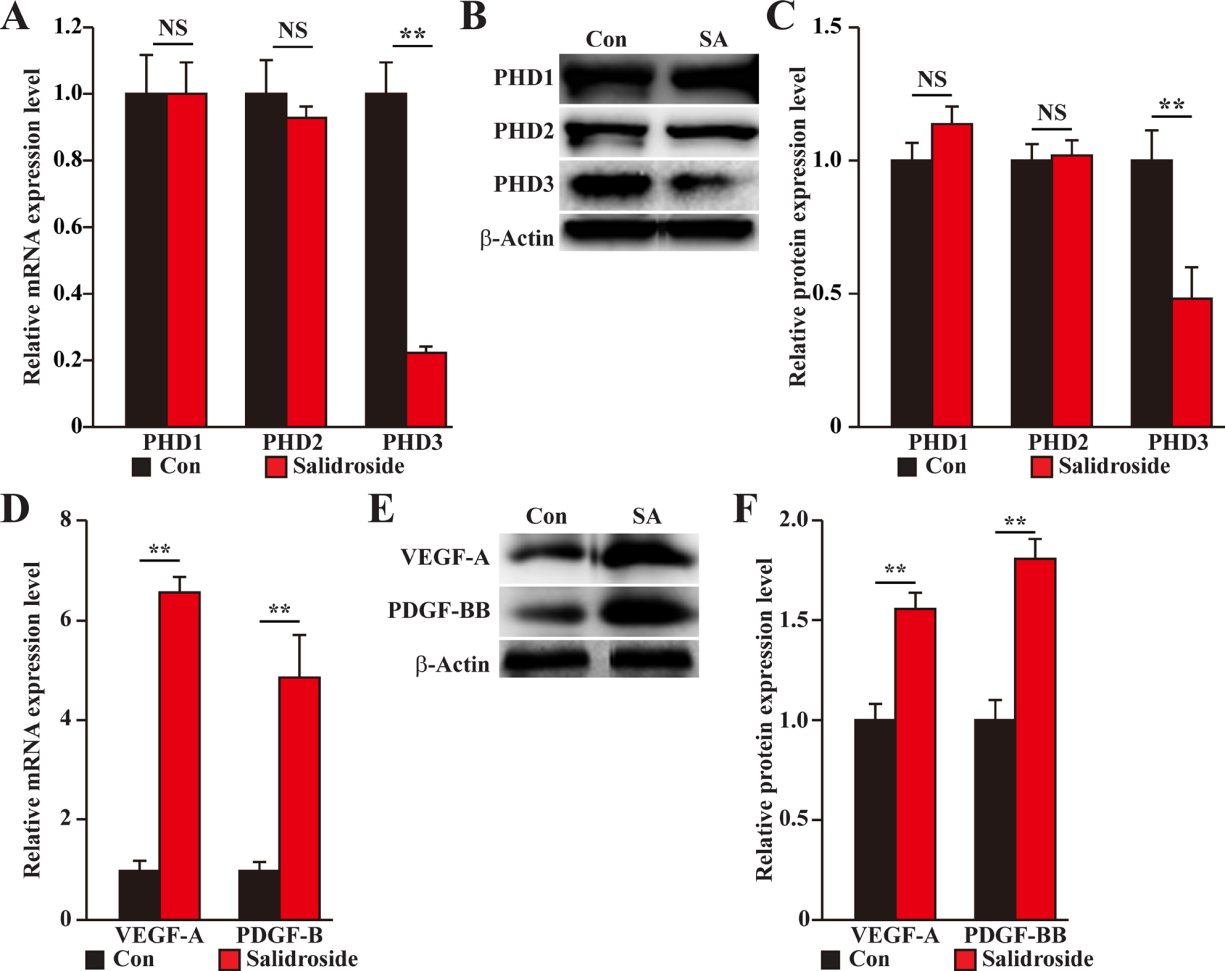
Salidroside promotes blood perfusion recovery by suppressing PHD3 and promoting angiogenic factors expression in the gastrocnemius muscle of diabetic HLI model mice (**A–C**) The expression levels of PHD family in the gastrocnemius muscle of the ischemic hind limb of diabetic HLI model mice treated with salidroside or PBS: (A) mRNA expression levels, as examined by quantitative RT-PCR (qPCR); (B) representative images of protein expression levels, as examined by western blotting; and (C) quantification of protein expression levels. (**D–F**) The expression levels of VEGF-A and PDGF-BB in the gastrocnemius muscle of the ischemic hind limb of diabetic HLI model mice treated with salidroside or PBS: (D) mRNA expression levels, as examined by qPCR; (E) representative images of protein expression levels, as examined by western blotting; and (F) quantification of protein expression levels. β-Actin was used for normalization in qPCR, and as a loading control in western blotting. Quantification data were shown as relative to that of control, and expressed as mean ± S.D. (*n* = 3 from three independent experiments). NS: not significant; ^**^P < 0.01; Con: animals treated with PBS; SA: animals treated with salidroside.

To elucidate the molecular mechanism behind the therapeutic angiogenesis effect of salidroside in the diabetic HLI model mice, we examined the expression levels of PHD family in the gastrocnemius muscle of the ischemic hind limb of mice treated with salidroside or PBS. We found that similar to the results using C2C12 cells, salidroside treatment specifically suppressed the expression levels of PHD3 (Figure 9A–9C). Concomitantly, the expression levels of VEGF-A and PDGF-BB were also enhanced (Figure 9D–9F). Together, these results clearly showed that salidroside might induce blood perfusion recovery in the ischemic hind limb of diabetic HLI model mice by inducing the expressions and secretions of VEGF-A and PDGF-BB through suppressing PHD3 accumulation. These angiogenic factors in turn affect the endothelial and smooth muscle cells, and subsequently, induces neoangiogenesis.

## DISCUSSION

Accumulative evidences showed that therapeutic angiogenesis might be a potential strategy for treating HLI. However, the angiogenesis potential, which is severely disrupted under hyperglycemia, is the largest obstacle for effective therapeutic angiogenesis in diabetic HLI patients [2, 31]; and as the consequence, the efficacy of therapeutic angiogenesis in diabetic patient is significantly less effective than in non-diabetic patient [6]. This occurs most plausibly due to the aberrant endogenous signaling pathways caused by hyperglycemic condition [2, 10, 11]. In this study, we found that hyperglycemia induces PHD3 protein accumulation in skeletal muscle cells, which in turn decreases HIF-1α protein accumulation, and subsequently impairs the paracrine functions of skeletal muscle cells. Although whether or not aberrant PHD3 expression could be observed in other cells involved in angiogenesis and impaired their angiogenesis function, our results clearly showed that treating skeletal muscle cells with PHD3 small molecule drug inhibitor, salidroside [14], as well as shRNA targeting PHD3, could induce HIF-1α protein accumulation. Furthermore, downregulation of skeletal muscle cells PHD3 effectively restores the paracrine function, as well as the proliferation and migration potentials of the skeletal muscle cells attenuated by hyperglycemia. Stimulation of skeletal muscle cells paracrine function enhances skeletal muscle cells–endothelial and/or smooth muscle cells communication, which subsequently promotes neoangiogenesis and blood perfusion recovery in the diabetic HLI model mice.

PHD family members have been known to play a central role in oxygen homeostasis by enhancing the degradation of HIF-1α protein, the master regulator of various angiogenic factors [16, 19, 32]. For these reasons, inhibition of PHD family has been considered as one of the potential strategies for therapeutic angiogenesis [15, 19, 20, 33–35]. Among the PHD family members, PHD3 is thought to be the primary regulator of HIF-1α under prolonged and severe hypoxia, owing to the presence of a feedback mechanism induced by the accumulation of HIF-1α [19, 21, 36–42]. Together with our finding showing that hyperglycemia induces PHD3 accumulation in skeletal muscle cells, these facts indicate that the upregulation of skeletal muscle cells PHD3 under ischemic, hyperglycemic conditions, which then affects the endothelial and smooth muscle cells, might be one of the most crucial reasons for the impaired angiogenic potential in the diabetic patient. We also show that targeting skeletal muscle cells PHD3 is effective to ameliorate the impaired blood perfusion recovery in diabetic HLI. Therefore, skeletal muscle cells PHD3 might be a potential target for therapeutic angiogenesis in diabetic HLI patients.

Salidroside (2-[4-hydroxyphenyl]ethyl beta-D-glucopyranoside) is the main compound of *Rhodiola* [43, 44], a plant that had been used in traditional medicines owing to its functions in promoting the adaptation potential to high altitude [45, 46]. Our data show that salidroside could specifically suppress skeletal muscle cells PHD3 induced by hyperglycemia, and enhance the expression and secretion of VEGF-A and PDGF-BB, which are essential for promoting both the migration and proliferation potentials of endothelial and smooth muscle cells [26–29]. Specific inhibition of PHD family is crucial for angiogenesis, as simultaneous depletion of multiple PHD genes, as showed by Taniguchi *et al.*, could lead to hepatotoxicity instead of improving vascular and muscular integrity [34]. However, due to their highly similar structure, most of the known PHD family inhibitors do not exert specificity towards PHD3 [47, 48]. Our results not only strongly suggest that salidroside might be a promising small molecule drug for diabetic HLI, but might also be a potential drug for other diseases, such as preeclampsia and severe anemia due to impaired iron uptake, in which aberrant PHD3 upregulation occurs [49, 50].

We also reveal that salidroside is able to enhance the proliferation and migration potentials of skeletal muscle cells, which are also suppressed by hyperglycemia. As mentioned above, skeletal muscle cells, through its secretory functions, is crucial for neoangiogenesis [13, 14], and thus, stimulating their proliferation might be one of the reasons for the angiogenic effect of salidroside under hyperglycemia. On the other hand, previous reports showed that cells forming blood vessels, *i.e.*, endothelial and smooth muscle cells, extend their pseudopodia and migrate toward angiogenic stimuli [26]. Thus, the reduced migration potential of skeletal muscle cells narrows the dissemination of skeletal muscle secretomes, while elevating this migration potential might promote the dissemination of angiogenic factors, and subsequently, enhance neoangiogenesis in larger area [14]. It is also noteworthy that while systemic administration of salidroside had been reported to improve the condition of blood glucose concentration [51], the localized administration of salidroside in our experiments did not affect the blood glucose concentration of the diabetic HLI model mice (Supplementary Table 1). These facts indicate that the therapeutic angiogenic effect achieved here is merely due to the neoangiogenesis effect of salidroside itself, but not due to the improved blood glucose condition.

Our *in vitro* and *in vivo* results showed that hyperglycemia-induced PHD3 accumulation suppressed the expressions and secretions of VEGF-A and PDGF-BB. VEGF-A is a critical factor for enhancing the growth and mobility of endothelial cells, and thus crucial for tube formation [28, 52]. Impaired VEGF-A expression under hyperglycemia had been reported, and is closely link with the dysfunction of endothelial cells [10, 11]. Hyperglycemia could also increase the production of advances glycation end products (AGE), which causes the aberrant expression of platelet-derived growth factor (PDGF)-BB. PDGF-BB, which was reported to be expressed by endothelial and skeletal muscle cells, could induce the migration of both endothelial cells and pericytes [14, 27, 29, 53, 54], and thus is crucial for the maturation of the newly formed blood vessels [12]. Efforts had been made to promote the expressions of VEGF-A and PDGF-BB for treating diabetic HLI [55–57], however, due to the complexity of the vascularization process, combination of angiogenic factors might be more potential for therapeutic angiogenesis [58, 59]. For these reasons, targeting PHD3 by salidroside might be a promising strategy, as it simultaneously regulates both VEGF-A and PDGF-BB.

Together, our findings demonstrate a molecular mechanism underlying the impaired angiogenesis potentials in the diabetic HLI, and identified skeletal muscle cells PHD3 as a potential therapeutic target. We also reveal that small molecule drug salidroside could also show its selectivity against skeletal muscle cells PHD3 under hyperglycemia. Furthermore, although whether or not salidroside affects other angiogenic factors and/or cells involved in angiogenesis under hyperglycemia remains to be elucidated, our findings demonstrated, for the first time, that salidroside could restore the secretions of VEGF-A and PDGF-BB from skeletal muscle cells under hyperglycemia, which in turn lead to the activation of cell–cell communication between skeletal muscle cells and endothelial and/or smooth muscle cells. The activation of cell–cell communication between skeletal muscle cells and endothelial and/or smooth muscle cells in turn induced neoangiogenesis, and subsequently, the blood perfusion recovery. Therefore, our present study proposes that intramuscular injection of salidroside might be a promising therapeutic strategy for diabetic HLI.

## MATERIALS AND METHODS

### Cell culture

The C2C12, HUVECs, and MOVAS cell lines were obtained from the American Type Culture Collection (ATCC). All cells were maintained in Dulbecco’s modified Eagle medium basic (Gibco, Life Technologies, Grand Island, NY) supplemented with 10% fetal bovine serum (FBS) (Biological Industries, Israel). The cell lines have been tested periodically for mycoplasma contamination using Mycoplasma Detection Kit-QuickTest (Biotool, Houston, TX). Cells were cultured under hypoxia by incubating them in the hypoxia chamber (Anaeropouch Box, 0.1% O2, Mitsubishi Gas Chemical, Tokyo, Japan) [15, 60], for 6 h or 12 h prior to RNA or protein extraction, respectively.

For hyperglycemia experiments, cells were cultured under the presence of 25 mM glucose with Hank’s Balance Salt Solution (Gibco) for 24 h prior to exposure to hypoxia [18]. Cells cultured the cells with DMEM culture medium with 5.5 mM glucose were used as control.

For salidroside treatment, cells were cultured with salidroside (purity ≥ 98%, Tauto Biotech, Shanghai, China, final concentration: 100 μg/ml) 24 h before exposure to hypoxia.

For knockdown experiments, cells were cultured in a 6-well plate and transfected with control vector (shCon) or PHD3-specific shRNA expression vectors using Lipofectamine 2000 (Invitrogen Life Technologies, Grand Island, NY) according to the manufacturer’s instruction. 24 h after transfection, the cells were treated with puromycin (final concentration: 2.5 μg/ml) for 48 h to eliminate untransfected cells.

For overexpression experiments, cells were transfected with the indicated vectors using Lipofectamine 2000 according to the manufacturer's instruction.

### Plasmids and constructs

Murine PHD3 (NM_028133.2) shRNA expression vectors (shPHD3-1 and shPHD3-2) and overexpression vector (pcPHD3) were constructed as described previously [14]. For shRNA expression vector, the specific target sites for murine PHD3 were predicted using the algorithm for predicting specific and high-efficiency RNAi target sites: shPHD3-1 (AGA TAT TTC TCT TTC TTG C) and shPHD3-2 (TTG TTA TGG ACG ATG AAC C) [60, 61]. A vector with a stretch of 7 thymines immediately downstream of the U6 promoter (shCon) was used as control.

### Experimental animals

Male C57BL/6 mice (8 weeks old) were purchased from the Third Military Medical University (Chongqing, China). Animal studies were carried out in the Third Military Medical University (Chongqing, China), and approved by the Laboratory Animal Welfare and Ethics Committee of the Third Military Medical University. All surgeries were performed under ketamine/xylazine anesthesia (intraperitoneal administration, 80 mg/kg body weight and 50 mg/kg body weight, respectively), and all efforts were made to minimize suffering.

### Diabetic HLI model

For inducing diabetic condition, mice were fed with high fat diet (20% kcal protein, 20% kcal carbohydrate, and 60% kcal fat) for 3 weeks prior to intraperitoneal injection of streptozotocin (Sigma Aldrich, St. Louis, MO; 40 mg/kg/day dissolved in sodium citrate buffer) for consecutive 5 days [30]. One week after the last injection, the mice were fasted overnight, and then blood glucose was assessed using an Accu-Check Integra (Roche Diagnostics, Shanghai, China) by tail vein puncture blood sampling. Those with blood glucose ≥ 16.7 mmol/L were considered to be diabetic and used for preparing diabetic HLI model mice model as described previously [15, 62, 63]. Briefly, the leg was shaved and depilated, and the proximal part of the left femoral artery was completely excised; while the right femoral artery was not excised and used as a control. Salidroside (100 mg/kg body weight dissolved in PBS) or PBS was administered intramuscularly into the gastrocnemius muscle one day after femoral artery excision and every 3 days thereafter. The mice were allocated randomly after surgery, and the investigator was blinded to the group allocation and during the assessment. Ischemic damage was visually assessed and scored as reported previously (0 = no difference from the non-ischemic hind limb, 1 = mild discoloration, 2 = moderate discoloration, 3 = severe discoloration, subcutaneous tissue loss, or necrosis, and 4 = amputation) at indicated time points [63].

### Laser doppler perfusion imaging

Mice were anesthetized as described above prior to the measurement of blood perfusion in both ischemic (left) and non-ischemic hind limbs (right) using Laser Doppler Perfusion Imaging (Moor Instruments Ltd, Axminster, Devon, England) at indicated time points. Blood perfusion ratio was obtained by normalizing the blood perfusion of ischemic hind limbs with that of the contralateral non-ischemic hind limbs as reported previously [12, 14, 15].

### Preparation of conditioned medium

C2C12 cells were cultured under hyperglycemia for 24 h as described above, treated with salidroside (final concentration: 100 μg/ml) for 24 h, washed, and then cultured further under hyperglycemia for 24 h. The culture medium was collected and filtrated through a 0.22 μm filter to obtain conditioned medium (CM-H/SA). For controls, cells were treated with PBS instead of salidroside for 24 h and cultured under low glucose or hyperglycemia for 24 h, and then the conditioned media (CM-L and CM-H, respectively) were collected as described above.

Conditioned media from C2C12 cells transfected with PHD3 overexpression plasmid, treated with salidroside and cultured under hyperglycemia; transfected with control plasmid, treated with salidroside and cultured under hyperglycemia; or transfected with control plasmid, treated with PBS and cultured under hyperglycemia were prepared as follows: C2C12 cells were transfected with pcPHD3 or pcDNA3.1(+) plasmid for 24 h, treated with 100 μg/ml salidroside or PBS, washed, and cultured under hyperglycemia as described above for 12 h. Then the conditioned media were collected as described above.

### RNA extraction and quantitative RT-PCR analysis

Total RNA from cells and gastrocnemius muscle was extracted with Trizol (Invitrogen Life Technologies) according to the manufacturer’s instruction, then 1 μg of the total RNA was reverse-transcribed into cDNA using the PrimeScript RT Reagent Kit with gDNA Eraser (Takara Bio, Dalian, China), and quantitative RT-PCR was performed using SYBR Premix Ex Taq (Takara Bio) to assess the mRNA expression levels. The sequences of the primers used for quantitative RT-PCR were shown in Supplementary Table 2. β-Actin was used to normalize sample amplifications. Results were shown as relative to the expression level in the corresponding controls, which are assumed as 1.

### Western blotting

Western blotting was performed as previously described [64]. For cell culture experiments, cells were collected and lysed with RIPA lysis buffer with protease inhibitor and phosphatase inhibitor cocktail (complete cocktail; Roche Applied Science, Mannheim, Germany). For mouse experiments, the gastrocnemius muscle was isolated and immediately homogenized with RIPA lysis buffer with protease inhibitor and phosphatase inhibitor cocktail (complete cocktail; Roche Applied Science) to obtain protein extracts. Equal amounts of the sample proteins were electrophoresed on sodium dodecyl sulfate polyacrylamide gel and transferred to a polyvinylidene fluoride (PVDF) membrane (Millipore, Billerica, MA). The antibodies used are listed in Supplementary Table 3, and the signal was measured by the SuperSignal West Femto Maximum Sensitivity Substrate detection system (Thermo Scientific, Waltham, MA). β-actin was used as a loading control. The quantitative analysis was performed using Quantity One (Thermo Scientific), and the results were shown as relative to the expression level in the corresponding controls, which are assumed as 1.

### Immunohistochemistry and immunofluorescence staining

Frozen gastrocnemius muscles were sectioned at 10 μm thickness using a cryostat and subjected to immunohistochemistry as described above [14].

For Ki67 staining, cells cultured under indicated condition and treated with salidroside or PBS were seeded in a 15-mm glass bottom cell culture dish, and then further cultured for 12 h. Cells were fixed with 4% paraformaldehyde and permeabilized for 5 min with PBS containing 0.1% Triton X-100. After blocking with 1% bovine serum albumin for 1 h, the samples were incubated at room temperature for 90 min with an anti-Ki67 antibody and subsequently, stained with secondary antibodies anti-rabbit Alexa Fluor 488 (Invitrogen Life Technologies). DAPI (Beyotime, Guangzhou, China) was used to stain nuclei. Images were taken with Microsystems-AF6000 (Leica, Heidelberg, Germany). For phalloidin staining, samples were incubated at room temperature for 30 min with phalloidin after blocking, and images were taken with Microsystems-TCS SP5 (Leica). The quantification of F-Actin formed from G-Actin polymerization was performed by fractal dimension as described previously [25], by using ImageJ software. For experiments with conditioned media, the cells were cultured with conditioned medium under hypoxia for 12 h prior to staining. The antibodies used were listed in Supplementary Table 2.

### Transwell chamber assay

Cells were cultured under hyperglycemia for 24 h prior to 24 h treatment with PBS or salidroside (final concentration: 100 μg/ml). Transwell chamber assay were then performed as described previously [14]. For experiments with conditioned media, conditioned media were placed in the lower chambers.

### Crystal violet staining

Cells were cultured under hyperglycemia for 24 h prior to 24 h treatment with PBS or salidroside (final concentration: 100 μg/ml) in a 96-well plate. Cells were fixed with 10% formalin for 5 min and stained with 0.05% crystal violet for 30 min.

### Hematoxylin and eosin staining

The gastrocnemius muscles were fixed with 4% paraformaldehyde for overnight prior to being embedded in paraffin and sectioned at 5 μm thickness using a cryostat. Sections were dewaxed using xylene and rehydrated before being stained with Hematoxylin and Eosin (Beyotime).

### Statistical analysis

Statistical analysis was performed using Student’s *t* test except for mouse experimental data, which were analyzed using a nonparametric Mann-Whitney test. A value of ^*^P < 0.05 was considered statistically significant, while a value of ^**^P < 0.01 was considered highly significant.

## SUPPLEMENTARY MATERIALS FIGURES AND TABLES



## Abbreviations

HLI: hind-limb ischemia
VEGF-A: vascular endothelial growth factor
PDGF: platelet derived growth factor
PDGF-BB: platelet derived growth factor-BB
PHD: prolyl hydroxylase domain
PHD3: prolyl hydroxylase domain 3
HIF-1α: hypoxia-inducible factor-1α
HUVECs: human umbilical vein endothelial cells
