# Supplementary material for: Elevating VEGF-A and PDGF-BB secretion by salidroside enhances neoangiogenesis in diabetic hind-limb ischemia
Source: Oncotarget. 2017 Oct 13;8(57):97187–205. doi: 10.18632/oncotarget.21907 (PMC5722555; doi:10.18632/oncotarget.21907)
Supplement: Supplementary file 1 [file oncotarget-08-97187-s001.pdf]

Elevating VEGF-A and PDGF-BB secretion by salidroside enhances neoangiogenesis in diabetic hind-limb ischemia

SUPPLEMENTARY MATERIALS

Supplementary Table 1: Blood glucose concentration in diabetic hind-limb ischemia model mice during the experiment

Control (mmol/L)					Salidroside (mmol/L)				
No	1 w HFD	3 w HFD	1 w after STZ (0 d post-surgery)	3 w post-surgery	No	1 w HFD	3 w HFD	1 w after STZ (0 d post-surgery)	3 w post-surgery
1	6.7	5.4	22.5	32.9	1	7.8	8.9	21.5	20.6
2	8.4	6.2	21	25.5	2	4.8	7.4	17.5	23.6
3	9.6	7.7	21.2	24.9	3	5.9	9.2	28.8	21
4	10.5	5.5	20.3	19.4	4	7.5	9.1	17.3	23.2
5	6.8	7.3	20	18.1	5	7.6	8.7	20	21.9
6	6	8.3	23.1	19.4	6	7.2	7.8	21.8	23.4
7	8.5	7.7	25.2	25.9	7	4.3	7.2	25.5	21
Mean	8.07	6.87	21.9	23.73	Mean	6.44	8.33	21.77	22.1
Stdev	1.65	1.16	1.83	5.20	Stdev	1.44	0.84	4.18	1.28

HFD: high fat diet; STZ: streptozotocin administration.

Supplementary Table 2: Primer pairs used for gene quantification by quantitative RT-PCR

Gene	Ref Seq No.	Forward	Reverse
VEGF-A	NM_001025257	GCAGAAGTCCCATGAAGTGAT	GTCTCAATCGGACGGCAGTAG
PDGF-B	NM_011057	AGCAGAGCCTGCTGTAATCG	GGCTTCTTTCGCACAATCTC
PHD1	NM_053208.4	GGAACCCACATGAGGTGAAG	ACCTTTCTGTCCCGATGCT
PHD2	NM_053207.2	GAAGCTGGGCAACTACAGGA	CATGTCACGCATCTTCCATC
PHD3	NM_028133.2	CAGGTTATGTTCGCCATGTG	CAGGACCCCTCCGTGTAAC
β-Actin	NM_007393	AGATGTGGATCAGCAAGCAG	GCGCAAGTTAGGTTTTGTCA

Supplementary Table 3: Antibodies and chemicals used for western blotting, immunohistochemistry, immunofluorescence, and phalloidin staining

Antibody	Product number	Maker	Experiment	Dilution
anti-VEGF-A	sc-152	Santa Cruz Biotechnology	Western Blotting	1/500
anti-PDGF-BB	Ab23914	Abcam	Western Blotting	1/1000
anti-PHD1	NB100-310	Novus Biological	Western Blotting	1/1000
anti-PHD2	NB100-138	Novus Biological	Western Blotting	1/1000
anti-PHD3	Ab184714	Abcam	Western Blotting	1/2000
anti-β-Actin	#4967	Cell Signaling Technology	Western Blotting	1/10000
anti-PECAM1	550274	BD Pharmingen	Immunohistochemistry	1/100
Monoclonal anti-murine α-SMA Cy3 conjugate	C6198	Sigma-Aldrich	Immunohistochemistry	1/100
Alexa Fluor 488 Goat Anti-Rat IgG	A11006	Invitrogen	Immunohistochemistry	1/100
anti-Ki67	Ab15580	Abcam	Immunofluorescence	1/300
Alexa Fluor 488 Donkey Anti- rabbit IgG	A21206	Invitrogen	Immunofluorescence	1/1000
Phalloidin	A34055	Invitrogen	Immunofluorescence	1/250
DAPI	C1006	Beyotime	Immunofluorescence	Not diluted

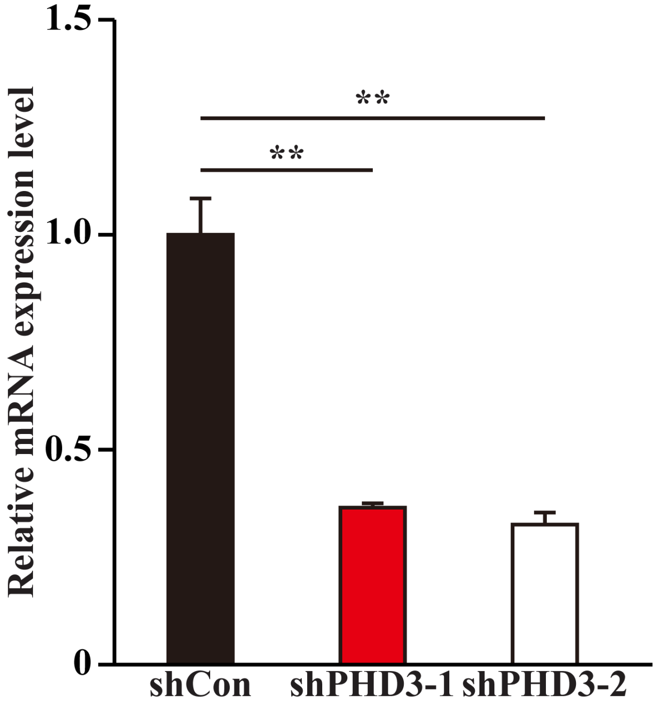

Supplementary Figure 1: The efficacies of short hairpin RNA (shRNA) expression vectors targeting skeletal muscle cells PHD3 under hyperglycemia. The PHD3 mRNA expression level in the C2C12 cells transfected with shPHD3s and cultured under hyperglycemia, as examined by quantitative RT-PCR. β-Actin was used for normalization. Cells transfected with shCon were used as control. Quantification data were shown as relative to control and expressed as mean ± S.D. (n = 3 from three independent experiments). $^{**}P < 0.01$.

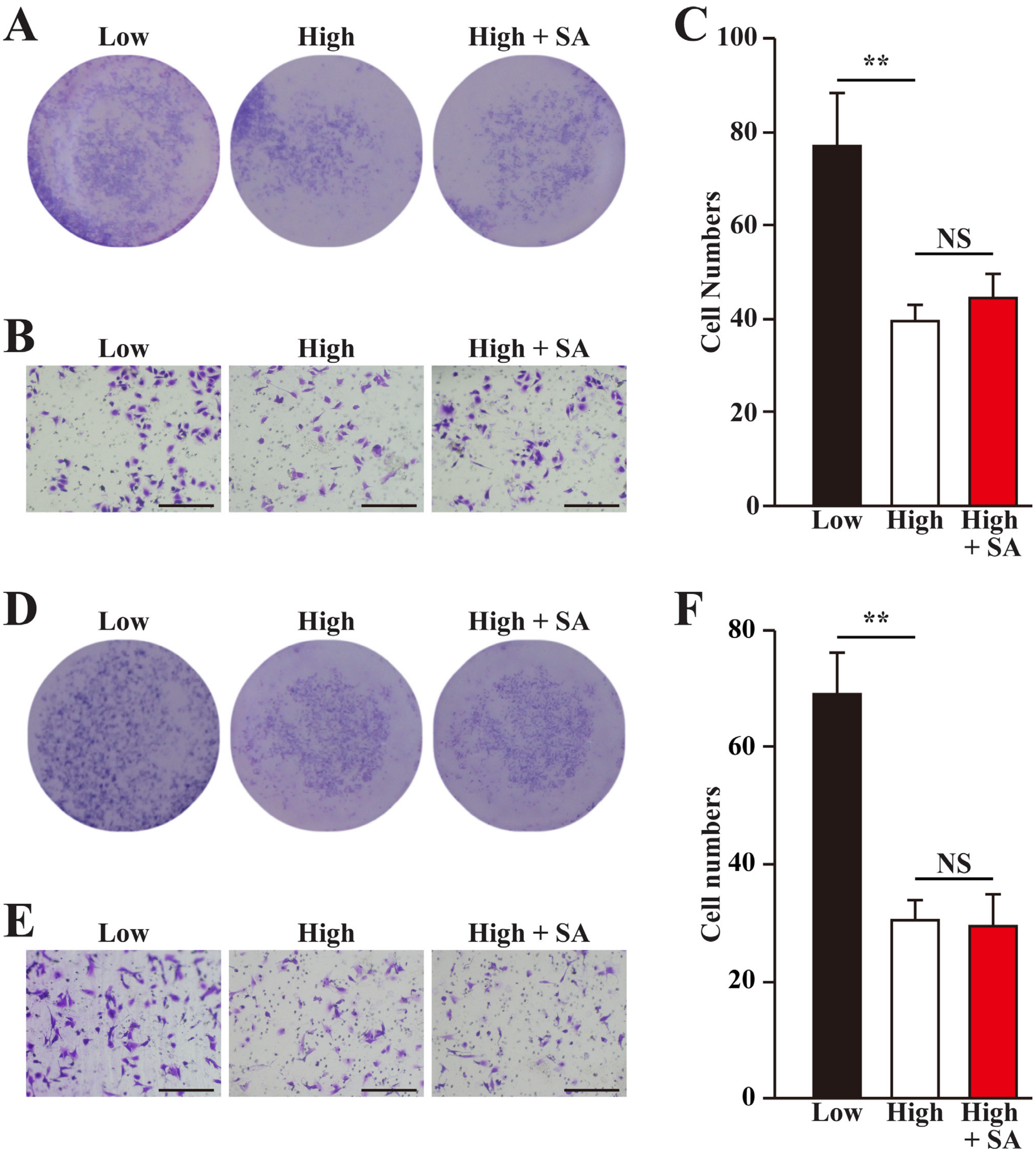

Supplementary Figure 2: The effects of salidroside on endothelial cells and smooth muscle cells under hyperglycemia. (**A**) The number of HUVECs cultured under hyperglycemia with or without salidroside, as analyzed using crystal violet. Representative images were shown. (**B, C**) The mobility of HUVECs cultured under hyperglycemia with or without salidroside, as examined using transwell chamber assay: (B) representative images (scale bars: 100 μm); and (C) quantification of migrated cells. (**D**) The number of MOVAS cells cultured under hyperglycemia with or without salidroside, as analyzed using crystal violet. Representative images were shown. (**E, F**) The mobility of MOVAS cells cultured under hyperglycemia with or without salidroside, as examined using transwell chamber assay: (E) representative images (scale bars: 100 μm); and (F) quantification of migrated cells. All experiments were done under hypoxia. Quantification data were expressed as mean ± S.D. ($n = 6$). $^{**}P < 0.01$; NS: not significant; Low: low glucose; High: high glucose; SA: salidroside.

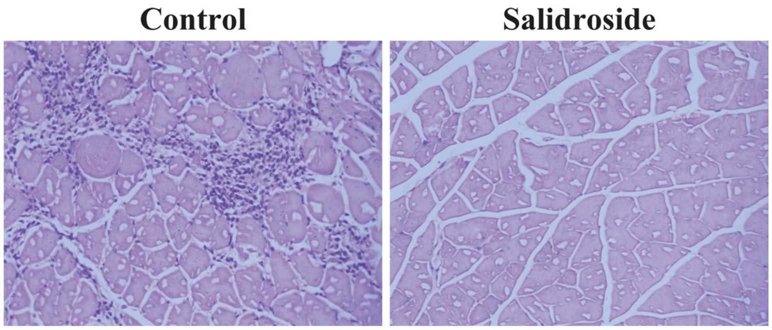

Supplementary Figure 3: The morphology of the gastrocnemius muscle tissue obtained from the ischemic hind limb of diabetic HLI mice treated with salidroside. The gastrocnemius muscle tissue obtained from the ischemic hind limb of diabetic HLI model mice treated with PBS (left panel) or salidroside (right panel) were stained using hematoxylin & eosin staining. Representative images were shown.
